# Tegument proteins of Epstein-Barr virus: Diverse functions, complex networks, and oncogenesis

**DOI:** 10.1016/j.tvr.2023.200260

**Published:** 2023-05-09

**Authors:** Takayuki Murata

**Affiliations:** Department of Virology, Fujita Health University School of Medicine, Toyoake, Japan

**Keywords:** EBV, Tegument, Envelopment, Egress, Infectivity, Oncogenesis

## Abstract

The tegument is the structure between the envelope and nucleocapsid of herpesvirus particles. Viral (and cellular) proteins accumulate to create the layers of the tegument. Some Epstein-Barr virus (EBV) tegument proteins are conserved widely in Herpesviridae, but others are shared only by members of the gamma-herpesvirus subfamily. As the interface to envelope and nucleocapsid, the tegument functions in virion morphogenesis and budding of the nucleocapsid during progeny production. When a virus particle enters a cell, enzymes such as kinase and deubiquitinase, and transcriptional activators are released from the virion to promote virus infection. Moreover, some EBV tegument proteins are involved in oncogenesis. Here, we summarize the roles of EBV tegument proteins, in comparison to those of other herpesviruses.

## Abbreviations

EBV: Epstein-Barr virus
IE: immediate-early
HSV: herpes simplex virus
VZV: varicella zoster virus
HCMV: human cytomegalovirus
HHV: human herpesvirus
KSHV: Kaposi's sarcoma-associated herpesvirus
NK: natural killer
PPIs: protein-protein interactions
BKRF4: *Bam*HI-K fragment rightward reading frame 4
CATC: capsid-associated tegument complex
CVSCs: capsid vertex-specific components
ORF: open reading frame
BCL2: B-cell lymphoma 2
UL/US: unique long/unique short
RL/RS: repeat long/repeat short
VP: virion protein
ICP: infected cell protein
gB: glycoprotein B
CDT1: chromatin licensing and DNA replication factor 1
NF-κB: nuclear factor-kappa B
IRF3: interferon regulatory factor 3
RIG-I: retinoic acid-inducible gene-I
cGAS: cyclic GMP-AMP synthase
TGN: trans-Golgi network
AP-1: activator protein 1
MAPK/MEK: mitogen-activated protein kinase
JNK: c-Jun N-terminal kinase
JAK/STAT: Janus kinase/signal transducer and activator of transcription
AGO2: Argonaute 2
CDK: cyclin-dependent kinase
PML-NB: promyelocytic leukemia protein-nuclear body
ND10: nuclear domain 10
ATRX: alpha thalassemia/mental retardation syndrome, X-linked
ERK: extracellular signal-regulated kinase
RSK: ribosomal S6 kinase
eIF4B: eukaryotic translation initiation factor 4B
USP7: ubiquitin-specific protease 7
ATF4: activating transcription factor 4
SRPK2: serine/arginine protein-specific kinase 2
MHV-68: murine gammaherpesvirus 68
TK: thymidine kinase
RR: ribonucleotide reductase
APOBEC3B: apolipoprotein B mRNA editing enzyme catalytic subunit 3B
LMP1: latent membrane protein 1
EBNA1: Epstein-Barr virus nuclear antigen 1
TNF-α: tumor-necrosis factor α
IL-6: interleukin 6
VEGF: vascular endothelial growth factor
RB: retinoblastoma
TERT: telomere reverse transcriptase
FGF: fibroblast growth factor
HK2: hexokinase 2
PKM2: pyruvate kinase 2
LDHA1: lactate dehydrogenase A1
GLUT1: glucose transporter 1
HLA: human leukocyte antigen
PD-L1: programmed cell death ligand 1

## 1 Introduction

Herpesviruses have double-stranded linear DNA genomes in icosahedral containers composed of viral capsid proteins. The nucleocapsid is wrapped in a lipid bilayer envelope, from which glycoproteins protrude. The space and/or the components between the nucleocapsid and envelope is termed the tegument [1]. The tegument of herpesviruses is mainly composed of viral proteins, with some cellular proteins. Tegument proteins do not comprise a solid structure like the nucleocapsid and, thus, a portion of the tegument structure can be released into the cytoplasm when the viral envelope fuses with the plasma membrane or endosomal membrane [2]. Some types of tegument proteins form interfaces with the exterior of the nucleocapsid (the inner tegument), and some others with membrane proteins on the interior surface of the envelope (outer tegument). Other tegument proteins form a complex network that connects the inner and outer tegument proteins.

Herpesvirus infections comprise latent and lytic phases [3,4]. In the latent phase, the virus exists as a circular double-stranded DNA molecule in the nucleus of a host cell, expressing only a small number of viral genes. This is an endurant type of infection, where the virus minimizes viral antigen presentation to avoid host immunity. By contrast, the lytic phase is an active type of infection, in which all viral genes are expressed, viral DNA is replicated, and progeny virus particles are produced. The lytic cycle starts with expression of a handful of viral genes, named immediate-early (IE) genes. IE genes are activators of viral gene expression and induce the expression of early viral genes. The proteins encoded by early genes, including those needed for nucleotide metabolism and DNA replication, amplify the viral genome in replication compartments in the host cell nucleus. The remaining viral lytic genes are categorized as late, and most encode viral structural proteins *i.e.*, capsid, tegument, and glycoproteins. Capsid proteins assemble into an icosahedral architecture, into which a viral genome is incorporated in the nucleus. Because the resultant nucleocapsid is too large to pass through nuclear pores, it must traverse the nuclear double membrane [5,6]. First, the nucleocapsid buds into the inner nuclear membrane (primary/initial envelopment). Some tegument proteins are attached to the exterior of the nucleocapsid in the nucleus before the initial envelopment. The temporal envelope fuses with the outer nuclear membrane (de-envelopment) to release nucleocapsid into the cytoplasm. The nucleocapsid buds into a cytoplasmic membrane structure (possibly derived from the TGN or endosome) [7]. The other tegument proteins are incorporated during this secondary envelopment. The cytoplasmic membrane structure finally fuses with the plasma membrane, and a mature progeny virion is released. The released virion attaches to a receptor on the plasma membrane and enters a cell. Two routes have been indicated for herpesvirus entry [8]. Route 1 involves fusion of the viral envelope with the plasma membrane and release of nucleocapsid and tegument proteins into the cytoplasm. In route 2, the virion is engulfed by an endosome, and the viral envelope fuses with the endosomal membrane to release nucleocapsid and tegument proteins. Released tegument proteins play a regulatory role in newly infected cells and promote virus infectivity. Other tegument proteins attached to the incoming nucleocapsid support nuclear transportation of the nucleocapsid. The linear viral DNA genome enters the nucleus, where the genome is circularized and gains histones to become an episome.

Herpesviruses are categorized as alpha-, beta-, and gamma-herpesviruses. To date, nine human herpesviruses have been identified. Herpes simplex virus type 1 (HSV-1), HSV-2, and varicella zoster virus (VZV) belong to the alpha-herpesvirus group. HSV-1 and 2 cause herpes labialis, keratitis, encephalitis, and genital herpes. Chicken pox in children is attributable to initial VZV infection, and the same virus later causes shingles. The beta-herpesviruses include human cytomegalovirus (HCMV), human herpesvirus type 6A (HHV-6A), HHV-6B, and HHV-7. HCMV causes interstitial pneumonia, retinitis, and congenital HCMV infection, and HHV-6B and 7 are agents of exanthema subitem. Epstein-Barr virus (EBV) and Kaposi's sarcoma-associated herpesvirus (KSHV) are gamma-herpesviruses. Initial EBV infection in children is associated with no obvious symptoms but can lead to infectious mononucleosis if initially infected during/after adolescence. Furthermore, EBV is associated with several cancers, such as Burkitt lymphoma, Hodgkin lymphoma, post-transplant lymphoproliferative disorder, T/NK cell lymphoma, chronic active EBV infection, nasopharyngeal carcinoma, and gastric carcinoma. KSHV is also an oncogenic virus, being the cause of Kaposi sarcoma, primary effusion lymphoma, and multicentric Castleman disease.

Some EBV tegument proteins are conserved across the Herpesviridae, but others—including BKRF4, BLRF2, and BNRF1—are unique to gamma-herpesviruses [9]. In this review, we summarize the structures and functions of EBV tegument proteins by comparison with other herpesviruses. Detailed electron microscopic analysis of the tegument structure of the whole EBV virion has not been performed, but protein-protein interactions (PPIs) analyses showed a plausible meshwork structure. Such complex PPIs hamper functional analysis of tegument proteins; following knockout of a tegument gene, loss of the encoded protein may affect incorporation of other tegument proteins. The multiple overlapping PPIs of tegument proteins may compensate for the loss of a tegument gene and obscure the phenotype of the mutant virus. Nevertheless, we and others have performed phenotypic analyses of EBV knockouts to assess the functions of tegument genes. We here summarize the findings of those analyses.

## 2 Composition of EBV tegument proteins

Mass spectrometry of purified EBV particles from culture medium detected some proteins homologous to the tegument proteins of alpha- and beta-herpesviruses [10]. Tegument proteins conserved across the Herpesviridae include BBLF1 (homolog of HSV UL11, HCMV UL99, and KSHV ORF38), BBRF2 (UL7, UL103, and ORF42), BGLF1 (UL17, UL93, and ORF32), BGLF2 (UL16, UL94, and ORF33), BGLF4 (UL13, UL97, and ORF36), BOLF1 (UL37, UL47, and ORF63), BPLF1 (UL36, UL48, and ORF64), BSRF1 (UL51, UL71, and ORF55), and BVRF1 (UL25, UL77, and ORF19) (Table 1). Because BGLF1 and BVRF1 are involved in viral-genome packaging, they are sometimes described as capsid proteins. However, BGLF1 and BVRF1 form the capsid-associated tegument complex (CATC; alternatively capsid vertex-specific components [CVSCs]) along with BPLF1, and so can be categorized as tegument components. Three tegument proteins, BKRF4 (homolog of KSHV ORF45), BLRF2 (ORF52), and BNRF1 (ORF75), are unique to gamma-herpesviruses (Table 1). KSHV homologs of these genes, ORFs45, 52, and 75, are incorporated into the tegument fraction [11,12]. Viral proteins that may be incorporated into the tegument include BALF1 (possibly related to KSHVORF16), BALF2 (homolog of HSV UL29, HCMV UL57, and KSHV ORF6), BGLF3.5 (homolog of HSV UL14 and KSHV ORF35, but not conserved in HCMV), BMRF1 (homolog of HSV UL42, HCMV UL44, and KSHV ORF59), BORF2 (UL39, UL45, and ORF61), BRLF1 (KSHV ORF50), BRRF2 (KSHV ORF48), and BXLF1 (HSV UL23 and KSHV ORF21) (Table 2). Because BDLF2 (homolog of KSHV ORF27) is a glycosylated type II membrane protein [13], it is likely to be an envelope, rather than a tegument, protein. Because some of these possible proteins, such as BALF2 and BMRF1, are abundantly expressed in cells, they may be incorporated into virions non-specifically. Several cellular proteins were also detected in EBV virions, such as ACTIN, TUBULIN, COFILIN, HSP70, and HSP90 [10].

**Table 1:** Tegument proteins of EBV.

EBV	alias name	HSV	HCMV	KSHV	Role in EBV lifecycle	Role in other herpesviruses
BBLF1	MyrP	UL11	UL99/pp28	ORF38	increase progeny production by improving egress [54,104], relocalize BGLF2 [51]	secondary envelopment [105]
BBRF2		UL7	UL103	ORF42	increase progeny production by improving infectivity [48], stabilize BSRF1 [48]	secondary envelopment, egress, and cell-to-cell spread [[100], [101], [102]]
BGLF1	CATC	UL17	UL93	ORF32	function not reported	cleavage/packaging [64]
BGLF2	MyrPBP	UL16	UL94	ORF33	increase progeny production by improving infectivity and egress [51,109], signal modification [[109], [110], [111], [112], [113]]	secondary envelopment, cell-to-cell spread [60,[121], [122], [123], [124]], nuclear egress [125,126]
BGLF4	vCDK	UL13	U97	ORF36	increase progeny production [142], late gene expression [[143], [144], [145]], initial envelopment [135]	viral gene expression, replication, progeny production, cell-to-cell spread [[148], [149], [150], [151]]
BOLF1	LTPBP	UL37	UL47	ORF63	increase progeny production by improving infectivity [41]	secondary envelopment [39,70,91], transport [[92], [93], [94]], deamidase activity [95,96]
BPLF1	LTP, CATC	UL36	UL48	ORF64	has de-Ub activity [76], increase viral DNA synthesis, progeny production [76,78,86], and oncogenesis [90]	has de-Ub activity [77], assembly, secondary envelopment [70], nuclear transportation, nuclear targeting of viral DNA [[71], [72], [73], [74], [75]]
BSRF1	PalmP	UL51	UL71	ORF55	increase progeny production [14], relocalize BBRF2 [48]	maturation, secondary envelopment, egress, and cell-to-cell spread [50,98]
BVRF1	CATC	UL25	UL77	ORF19	function not reported	packaging [65,66], nuclear egress [36,68,69], and uncoating [67]
BKRF4				ORF45	increase progeny production by improving infectivity [57], inhibit DDR [159,160], relocalize BGLF2 [57]	involved in replication [164], assembly, envelopment, egress [165,166], transport [167], signal modification [[168], [169], [170], [171], [172], [173], [174], [175], [176]]
BLRF2				ORF52	increase progeny production by improving infectivity [22]	increase progeny production by improving infectivity [179], inhibit cGAS [[180], [181], [182]]
BNRF1	MTP			ORF75	increase infectivity by increasing viral gene expression, especially in primary B cells [62]	disrupt ATRX and increase viral gene expression [158]

The numbers in the “Roles” column indicate reference numbers.

**Table 2:** Possible tegument proteins of EBV.

EBV	alias name	HSV	HCMV	KSHV	Role in EBV lifecycle	Role in other herpesviruses
BALF1	vBCL2			(ORF16)	inhibit apoptosis [198]	viral gene expression, replication, progeny production, inhibit apoptosis in KSHV [200]
BALF2	ssDNABP	UL29	UL57	ORF6	essential for viral DNA synthesis, transcriptional activator [201,202]	essential for viral DNA synthesis [9]
BGLF3.5		UL14		ORF35	no obvious function [188]	increase progeny production in HSV [16,190]
BMRF1	EA-D	UL42	UL44	ORF59	essential for viral DNA synthesis [201,205]	essential for viral DNA synthesis [9]
BORF2	RR	UL39	UL45	ORF61	increase progeny production [214], increase P53 [108], inhibit APOBEC3B [214,215], involved in nucleotide metabolism [9]	increase progeny production in non-dividing cells [218], involved in nucleotide metabolism, inhibit APOBEC3B in HSV [219]
BRLF1	Rta			ORF50	transcriptional activator [222], stabilize BORF1 [19]	transcriptional activator in KSHV [223]
BRRF2				ORF48	increase progeny production [224]	not reported
BXLF1	TK	UL23		ORF21	no obvious function in cultured cell [211], nucleotide biosynthesis [210]	increase progeny production by improving infectivity in KSHV [213], nucleotide biosynthesis

The numbers in the “Roles” column indicate reference numbers.

Note that all of the 12 tegument proteins in Table 1 and 4 out of the 8 possible tegument proteins in Table 2 were readily detected by mass spectrometry in virion [10], although BALF1, BGLF3.5, BRLF1, and BRRF2 proteins in Table 2 were below the threshold. BALF1 is a viral homolog of the anti-apoptotic protein, BCL2. Although not identified as a tegument protein, BALF1 is included in this table because overexpressed BALF1 protein was detected in EBV virions by western blotting [14], as was KSHV vBCL2 (ORF16) [15]. BGLF3.5 has not been detected in EBV virion, but its homologs HSV UL14 [16,17] and KSHV ORF35 [18] are identified in virions. Endogenous BRRF2 protein was detectable by western blotting of EBV virion using our antibody [14], and its KSHV homolog ORF48 protein is also identified by mass spectrometry in the virion fraction [18]. BRLF1 protein is recently identified in EBV virion fraction by western blotting [19], and the KSHV homolog ORF50 is detected by western blotting of KSHV virion proteins [11].

## 3 PPI network of EBV tegument proteins, and with nucleocapsid and envelope proteins

**Fig. 1:**
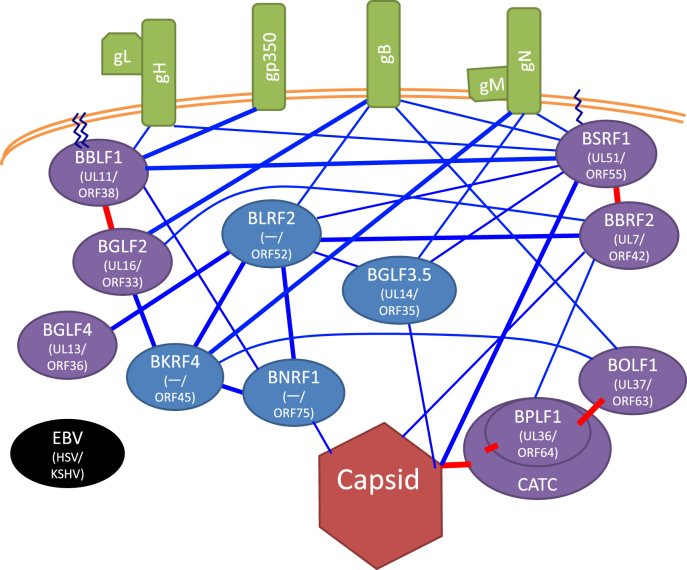
Simplified EBV virion protein network. Network formed by protein-protein interactions (PPIs) of tegument proteins (oval), nucleocapsid (dark red hexagon at bottom), and envelope proteins (green box). Purple and blue ovals, conserved and non-conserved tegument proteins, respectively. EBV gene names together with those of their homologs in HSV and KSHV are noted. Saw-toothed line, post-translational lipid modifications—palmitoylation and myristoylation. A copy of BGLF1, two copies of BVRF1, and two copies of BPLF1 comprise the capsid-associated tegument complex (CATC). Red lines indicate interactions conserved across herpesviruses. Thick lines indicate interactions that were previously reported in at least one species of human herpesviruses in addition to EBV, or that were found only in EBV but validated by two or more methods. (For interpretation of the references to colour in this figure legend, the reader is referred to the Web version of this article.)

**Fig. 2:**
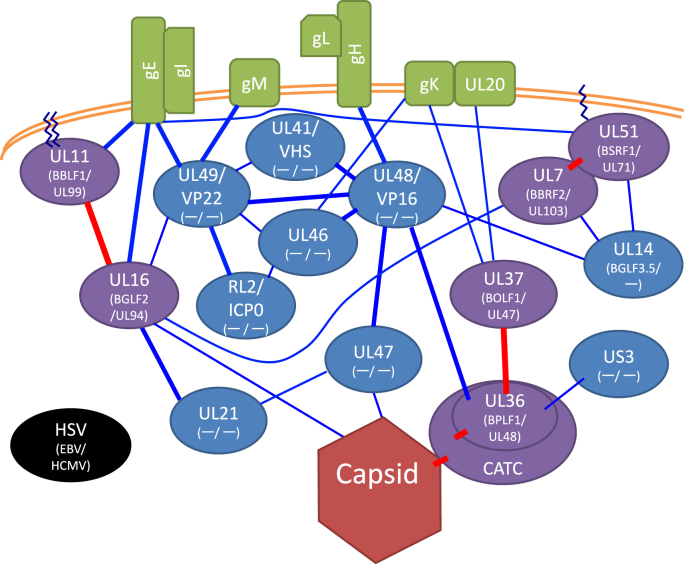
Simplified HSV virion protein network. Network formed by protein-protein interactions (PPIs) of tegument proteins (oval), nucleocapsid (dark red hexagon at bottom), and envelope proteins (green box). Purple and blue ovals, conserved and non-conserved tegument proteins, respectively. HSV gene names together with those of their homologs in EBV and HCMV are shown. Saw-toothed line, post-translational lipid modifications—palmitoylation and myristoylation. A copy of UL17, two copies of UL25, and two copies of UL36 comprise the CATC. Red lines indicate interactions conserved across herpesviruses. Thick lines indicate interactions that were previously reported in more than one papers. (For interpretation of the references to colour in this figure legend, the reader is referred to the Web version of this article.)

Tegument proteins form a complex PPI network that attaches the viral envelope to the nucleocapsid. We prepared a simplified diagram of the EBV PPI network based on prior reports [[20], [21], [22], [23]] (Fig. 1). For comparison, a PPI network centering on HSV tegument proteins was also generated (Fig. 2) [2,7,[24], [25], [26], [27]]. Most PPIs have been detected in lysates (by immunoprecipitation) or cells (yeast two-hybrid or complementation assay of split marker), but not in the tegument. Nevertheless, these interactions are important because layers of tegument proteins are formed during envelopment in cells. Unfortunately, no similar schema for HCMV is available possibly because of the large number of tegument components [[28], [29], [30]].

A significant number of EBV tegument proteins are not conserved in subfamilies other than gamma-herpesviruses (blue ovals in Fig. 1), and many HSV tegument components are not present in other subfamilies (blue ovals in Fig. 2). So, the gamma-herpesviruses are likely to have evolved separately from the alpha- and beta-herpesviruses, and the tegument composition may reflect cell tropism, mode of infection, and disorders. For instance, the HSV and HCMV teguments have powerful transcriptional activators of IE genes (UL48/VP16 and UL82/pp71, respectively), but there is no counterpart of these genes in EBV. Their absence may explain the typically latent form of infection with EBV.

The nucleocapsid vertex-CATC-BOLF1/UL37-membrane protein axis PPI is preserved across subfamilies (Fig. 1, Fig. 2). Indeed, high-resolution cryogenic electron microscopy of EBV [31,32] showed that the capsid vertex-tegument interface encompasses CATC. Interestingly, EBV CATC, composed of BGLF1, BVRF1, and BPLF1, crowns more efficiently at the portal vertex rather than penton vertices, suggesting a more important role for CATC in EBV genome containment. CATC infrequently binds to penton vertices of KSHV [33], but HSV-1 CATC occupies most portal and penton vertices [34]. The HCMV nucleocapsid is associated with fewer copies of CATC at penton vertices, but its pentons and hexons are fixed by a unique tegument protein, UL32/pp150 [35]. The binding of BPLF1 and BOLF1 homologs is highly conserved in the family (Fig. 1, Fig. 2) [21,27,[36], [37], [38]], suggesting the importance of their interaction. BOLF1 links to glycoprotein B (gB) (Fig. 1) [21], whereas HSV-1 UL37 interacts with gK and UL20 (Fig. 2) [39]. EBV BPLF1 is linked to BBRF2 [40], BOLF1 associates with BKRF4 (Fig. 1) [41], and HSV-1 UL36 binds to UL48 [42] and US3 [43] (Fig. 2).

The complex of two tegument proteins, BSRF1 and BBRF2, is important in the EBV lifecycle, because these have homologs in other herpesviruses [14,44,45]. The UL51 protein of HSV-1 is post-translationally modified by palmitoylation [46], which facilitates its membrane association. Although palmitoylation of EBV BSRF1 and HCMV UL71 proteins has not been confirmed, it is likely because the modified N-terminal cysteine is conserved and associated with membranes, particularly of the Golgi apparatus [14,47]. EBV BSRF1 associates with many viral proteins, besides BBRF2, including gB, gH [40], gN, BLRF2 [22], and BGLF3.5 [14] (Fig. 1). BBRF2 associates with BGLF2 [48], BLRF2, BPLF1, and BcLF1 (major capsid protein [MCP]) [40] (Fig. 1). The HSV UL51 protein binds to UL14 [49] and gE [50]. The binding of UL7 to UL14 and UL16 proteins has been reported (Fig. 2) [11].

Another well-conserved tegument interaction is that of the EBV BBLF1-BGLF2 complex (Fig. 1) [51]; interactions of HSV UL11-UL16 [52] and HCMV UL99-UL94 [53] have also been reported. BBLF1 and its counterparts are post-translationally modified by palmitoylation and myristoylation [[54], [55], [56]]. BBLF1 is a small protein, which interacts with BNRF1, gH and gp350 [[20], [21], [22]]. BGLF2 associates with BBRF2, BKRF4, and gB (Fig. 1) [21,22,48,57]. HSV UL11 binds with gE [58], and UL16 binds to gE [59], UL49 [60], UL7 [21], UL21 [61], and UL35 (small capsid) [21] (Fig. 2).

HSV UL21, 41, 46, 47, 48, 49, and RL2 are present only in alpha-herpesviruses. In EBV, the loss of these tegument proteins can be compensated for by gamma-herpesvirus-specific proteins, BKRF4, BNRF1, and BLRF2. BKRF4 is linked to BGLF2, BLRF2, BOLF1, BNRF1, and gN [22,41,57]. BNRF1 interacts with tegument proteins, BKRF4, BBLF1, and BLRF2, and possibly BFRF3 (small capsid) [[20], [21], [22]]. We reported that BNRF1 interacts with capsid proteins [22], and the suppression by BNRF1 gene knockout of nucleocapsid nuclear transport in B cells also suggests an interaction with the nucleocapsid [62]. BLRF2 binds to many tegument proteins, BGLF4, BKRF4, BNRF1, BSRF1, BBRF2, BGLF3.5, and to gB [20,22], and is thus hypothesized to be a tegument hub [22].

EBV BGLF3.5 is a homolog of HSV UL14 but has no counterpart in HCMV. It interacts with BSRF1, as HSV UL14 interacts with UL51 [14,22]. It also associates with BLRF2 and BBRF1, the portal protein [22]. BGLF4 is linked to BLRF2 [22].

**Fig. 3:**
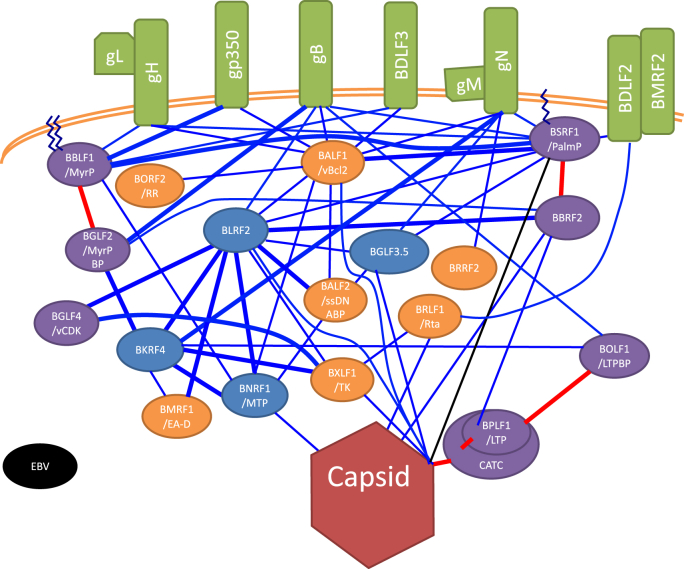
Detailed EBV virion protein network. Network formed by protein-protein interactions (PPIs) of all tegument proteins (oval), nucleocapsid (dark red hexagon at the bottom), and envelope proteins (green box). Purple and blue ovals, conserved and non-conserved tegument proteins, respectively. Orange ovals show possible tegument components not included in Fig. 1. EBV gene names are provided in ovals. Saw-toothed line, post-translational lipid modifications—palmitoylation and myristoylation. A copy of BGLF1, two copies of BVRF1, and two copies of BPLF1 comprise the capsid-associated tegument complex (CATC). Red lines indicate interactions conserved across herpesviruses. Thick lines indicate interactions that were previously reported in at least one species of human herpesviruses in addition to EBV, or that were found only in EBV but validated by two or more methods. (For interpretation of the references to colour in this figure legend, the reader is referred to the Web version of this article.)

The detailed PPIs of EBV tegument components are shown in Fig. 3 [[20], [21], [22]]. EBV BALF1 is a homolog of the anti-apoptotic host protein, BCL2. It associates with multiple membrane (gH, gP350, gB, BDLF3, and gN), tegument (BLRF2, BNRF1, BALF2, BSRF1, and BORF2), and capsid proteins (BBRF1) [14,[20], [21], [22]]. Being a type II membrane protein, BDLF2 has a transmembrane domain and forms a complex with an envelope protein, BMRF2 [13,63]. It also interacts with BSRF1 and BRLF1 [22]. BALF2 and BMRF1 are required for lytic viral genome DNA synthesis and may be incorporated into virions via interactions with other tegument proteins (BALF1, BLRF2, BNRF1, and BGLF3.5 for BALF2; BKRF4, and BLRF2 for BMRF1) [22]. BRLF1 is associated with BDLF2, BXLF1, and a capsid component, BORF1 [19,20,22]. BXLF1 is linked to BKRF4, BGLF4, BLRF2, BRLF1, and a capsid protein, BBRF1 [22]. BORF2 and BRRF2 each have one interacting partner, BALF1 and gN, respectively [20,22].

## 4 Roles of EBV tegument proteins in the virus lifecycle

### 4.1 The CATC: BGLF1 (homolog of HSV UL17, HCMV UL93, and KSHV ORF32), BVRF1 (UL25, UL77, and ORF19), and BPLF (UL36, UL48, and ORF64)

The EBV CATC, composed of one BGLF1, two BVRF1, and two BPLF1 proteins, fully occupies the five sitting sites at the portal vertex of the EBV nucleocapsid, whereas up to two CATCs occupy the five available CATC-binding sites at penton vertices [31,32]. In HSV, not only the portal but also the penton vertices are fully crowned by CATC [34]. So, the role of EBV CATC may be slightly different from that of HSV CATC, at least in terms of structural dynamics. Still, it must be emphasized that the CATC of all herpesviruses links the nucleocapsid and tegument proteins.

The roles of EBV BGLF1 and BVRF1 are unclear, but can be inferred from other herpesviruses, such as HSV-1. HSV UL17 (homolog of BGLF1) is essential for viral genome DNA cleavage and packaging [64]. UL25 (homolog of BVRF1) is required for packaging but not for cleavage [65,66]. Interestingly, the UL25 is also involved in viral genome uncoating immediately after infection [67] and nuclear egress of the nucleocapsid via interaction with the nuclear egress complex [36,68,69]. So, EBV BGLF1 and BVRF1 may be involved in viral genome cleavage and packaging, and possibly also viral genome containment, nuclear export, and nucleocapsid uncoating.

EBV BPLF1 and its homologs have been investigated extensively. HSV UL36 links nucleocapsid and tegument proteins. Indeed, knockout of the UL36 gene in HSV-1 caused cytoplasmic accumulation of unenveloped nucleocapsids, implicating UL36 in secondary envelopment [70]. In addition, HSV-1 UL36 is reported to play multiple roles in nuclear transport and viral DNA release into the nucleus [[71], [72], [73], [74], [75]]. The N-terminal part of BPLF1 encodes de-ubiquitination and de-neddylation activities, which are conserved across the family [76,77]. BPLF1 reduces the activity of viral ribonucleotide reductase [78], targets polymerase eta and Rad18 and deregulates the DNA damage tolerance pathway to increase progeny production [[79], [80], [81]], promotes viral replication by inducing accumulation of the licensing factor CDT1 [76], inhibits cullin-RING ligases [82], targets topoisomerase II [83], downregulates innate immunity (NF-κB and IRF3 pathways) [[84], [85], [86], [87]], and inhibits selective autophagy by targeting the autophagy receptor [88]. Caspase-1 cleaves BPLF1 and enhances nuclear localization of catalytically active BPLF1 during the lytic cycle [89]. Because of these functions, knockdown or knockout of BPLF1 markedly reduces viral DNA synthesis and progeny production [76,78,86,90]. Interestingly, BPLF1-disrupted EBV exhibited reduced B-cell transformation activity in cell culture and suppressed tumor formation in a humanized model [90].

### 4.2 BOLF1 (homolog of HSV UL37, HCMV UL47, and KSHV ORF63)

To date, only one study has focused on the role of EBV BOLF1 [41]. Disruption of BOLF1 had little or no effect on viral gene expression or DNA replication. The knockout virus produced progeny at a level similar to the wild-type, but the progeny had reduced infectivity.

HSV UL37, a homolog of BOLF1, is implicated in secondary envelopment [70]. UL37 mediates the interaction of CATC with the gK/UL20 complex on the membrane, both of which are important for secondary envelopment [39,91]. It is also involved in nucleocapsid transport [[92], [93], [94]]. Also, UL37 has deamidase activity, by which it blocks antiviral responses by RIG-I and cGAS [95,96]. By contrast, KSHV ORF63 suppresses innate immunity by inhibiting inflammasome signaling [97].

### 4.3 BSRF1 (homolog of HSV UL51, HCMV UL71, and KSHV ORF55)

BSRF1 is post-translationally modified by palmitoylation and associates with viral envelope or membrane. The phenotype of BSRF1-knockout EBV was indistinguishable from wild-type EBV in HEK293 cells, but knockdown of BSRF1 in B95-8 cells significantly decreased infectious progeny production [14]. Expression of BSRF1 promoted BBRF2 transport from the nucleus to the cytoplasm [48].

HSV UL51 is also modified by palmitoylation, which is required for its membrane localization [46]. UL51 is implicated in viral maturation, secondary envelopment, egress, and intercellular spread [50,98]. UL51 is needed for proper localization of UL7 to cytoplasmic membranes [45]. It also associates with gE and UL14, which promote proper localization and envelopment, respectively [49,50]. In addition, UL51 phosphorylation affects viral nuclear egress and intercellular spread [99].

### 4.4 BBRF2 (homolog of HSV UL7, HCMV UL103, and KSHV ORF42)

Knockout of the EBV BBRF2 gene reduced progeny production without affecting viral gene expression and DNA replication [48]. The number of progeny produced was not reduced, but their infectivity was decreased. Co-expression of BBRF2 protected its interacting partner, BSRF1, from proteasome-dependent degradation, and co-expression of BSRF1 is needed for proper localization of BBRF2 [48], indicating the importance of the PPI.

Loss of the UL7 gene, a homolog of BBRF2, in HSV results in reduced virus replication and smaller plaques [[100], [101], [102]]. A UL7 deletion mutant of pseudorabies virus, an animal alpha-herpesvirus, had defects in secondary envelopment and egress [103].

### 4.5 BBLF1 (homolog of HSV UL11, HCMV UL99, and KSHV ORF38)

BBLF1 is a membrane-associated protein modified by palmitoylation and myristoylation [54]. Myristoylation of BBLF1 is crucial for its stabilization and localization to the TGN [54]. BBLF1 interacts with BGLF2, thereby mediating proper cytoplasmic localization of BGLF2 to the TGN [51]. BBLF1 knockdown reduced EBV progeny production [54]. We found that, rather than being secreted extracellularly, BBLF1-null mutant virus progeny remained associated with the host cell. Because BBLF1 knockout and wild-type viruses produced similar numbers of infectious progeny in total, BBLF1 is likely to promote extracellular egress without affecting processes at virion maturation [104].

All EBV BBLF1 homologs in other herpesviruses undergo palmitoylation and myristoylation. HSV UL11 is involved in secondary envelopment [105] and so knockout of the encoding gene reduces progeny production and causes cytoplasmic accumulation of unenveloped nucleocapsids. Its associations with UL16 and gE are implicated in secondary envelopment [58,106,107].

### 4.6 BGLF2 (homolog of HSV UL16, HCMV UL94, and KSHV ORF33)

EBV BGLF2 arrests the cell cycle at G_1_/S phase by inducing p21 [108]. It also induced AP-1 signaling by activating the P38 MAPK and JNK signaling pathways, thereby promoting EBV lytic replication [109,110]. In addition, BGLF2 inhibits NF-κB [111] and interferon-induced JAK/STAT pathway [112,113] signaling, to subvert antiviral innate immunity and induce the viral lytic cycle. It was also reported to de-regulate miRNA functions by targeting AGO2 [114]. BGLF2 is incorporated not only into virions but also exosomes, and exosomal BGLF2 facilitates *de novo* EBV infection [115]. Disruption of the BGLF2 gene had little effect on viral gene expression and DNA synthesis, but extracellular production of infectious progeny was suppressed by about 10-fold and the cell-associated progeny level was also decreased [51,109]. So, the BGLF2 gene improves not only virus egress but also the infectivity of secreted virions [109]. BGLF2 interacts with BBLF1 and BKRF4 [51,57]. When expressed alone, BGLF2 localizes to the nucleus and cytoplasm, but its co-expression with BBLF1 recruited BGLF2 to the TGN [51], and addition of BKRF4 re-localized BGLF2 to the nucleus and perinuclear region [57]. MHV-68 ORF33 is associated not only with cytoplasmic but also with nuclear nucleocapsids [116], suggesting that BGLF2 associates with nucleocapsids in the nucleus before primary envelopment. Other reports on KSHV ORF33 suggest roles for BGLF2 in the cytoplasm [117,118].

UL16-null mutant of HSV-1 exhibits about a 10-fold reduction in virus yield [119], and that of HSV-2 has 50-100-fold progeny loss [120]. Disruption of the UL16 gene in HSV-1 is associated with cytoplasmic accumulation of nucleocapsids [60]. HSV-1 UL16 is localized to the nucleus and cytoplasm, and interacts with nucleocapsid [121,122] and membrane proteins, including UL11 [123] and gE [59]. These findings, in conjunction with information from other herpesviruses, implicate UL16 in secondary envelopment and intercellular spread [124]. However, it must also be noted that HSV-2 UL16 plays an important role in the nuclear egress of nucleocapsids [125,126], suggesting that the roles of UL16 homologs are different depending on the virus species. In addition, modification of signaling pathways, *e.g.*, MAPK, NF-κB, and JAK/STAT, has not been reported for HSV UL16 or KSHV ORF33.

### 4.7 BGLF4 (homolog of HSV UL13, HCMV UL97, and KSHV ORF36)

The BGLF4 gene encodes a serine/threonine protein kinase conserved across the family Herpesviridae, which has substrate similarity with host cyclin-dependent kinase (CDK). It phosphorylates many viral proteins, such as BGLF4, BMRF1, EBNA1, EBNA2, BXLF1, and BGLF2 [[127], [128], [129], [130]], as well as host proteins, such as EEF1D, P27, CHK1, RAD51, TIP60, and SAMHD1 [[131], [132], [133], [134]]. It induces disassembly of nuclear lamina [135], regulates microtubule dynamics [136], suppresses IRF3 signaling [137], mediates premature condensation of chromosomes [138], blocks host chromosomal DNA replication [139,140], and modulates nuclear pore complexes [141]. Compared to wild-type virus, progeny production by BGLF4-knockout EBV was reduced by about an order of magnitude [142]. The BGLF4 gene may modulate the expression of some late genes and initial envelopment at the nuclear membrane [[143], [144], [145]]. Furthermore, the BGLF4 gene, but not its thymidine kinase BXLF1, is needed for inhibition of virus replication by ganciclovir or acyclovir [146].

HSV UL13 phosphorylates substrates targeted by host CDKs, like BGLF4 [147], and plays roles in the expression of a subset of viral genes, viral replication, progeny production, intercellular spread, and evasion of host immunity [[148], [149], [150], [151]].

### 4.8 BNRF1 (homolog of KSHV ORF75)

The major tegument protein BNRF1 is conserved only among gamma-herpesviruses. It is expressed in latent cells, too, and is targeted by CD4^+^ and CD8^+^ T cells [152,153]. Disruption of the BNRF1 gene had little effect on EBV replication and progeny production. However, upon infection to primary B cells, viral gene expression was significantly repressed by the disruption, thereby suppressing B-cell transformation [62]. The group therefore speculated that nuclear transport of the nucleocapsid was inhibited by disruption of the BNRF1 gene. Later, however, other group showed that BNRF1 is involved in transcriptional activation of viral genes upon infection; BNRF1 targets PML-NB (also known as ND10) and disrupts the antiviral histone chaperone complex Daxx-ATRX, thereby increasing the expression of viral genes immediately after infection [154,155]. In addition, BNRF1 destabilizes the SMC5/6 cohesin complex to increase viral DNA replication [156] and induces centrosome amplification and thus chromosomal instability [157].

KSHV ORF75 disrupts ATRX to antagonize PML-NB-mediated intrinsic immunity, thus inducing viral gene expression [158]. Also, ORF75-null KSHV fails to produce IE genes, making ORF75 essential for viral replication, unlike EBV BNRF1.

### 4.9 BKRF4 (homolog of KSHV ORF45)

BKRF4 is an EBV late phosphoprotein [57] conserved among gamma-herpesviruses, albeit at low similarity. Knockout of the BKRF4 gene decreased progeny levels, possibly by reducing infectivity, but had no effect on viral gene expression and DNA replication [57]. Upon lytic induction of infected cells, BKRF4 localizes to the nucleus and peri-nuclear region. When expressed alone, BKRF4 and BGLF2 localize to the nucleus and cytoplasm, respectively, and co-expression of BKRF4 with BGLF2 re-localizes BGLF2 to the nucleus [57]. Interestingly, BGLF2 activates AP-1 signaling, an effect repressed by co-expression of BKRF4 [109]. BKRF4 associates with many other tegument proteins (Fig. 3), suggesting it to be a hub in the tegument meshwork [22]. BKRF4 inhibits the DNA damage response, suggesting involvement in oncogenesis [159]. Mutagenesis and structural analyses revealed that BKRF4 inhibits the DNA damage response by binding to partially unfolded nucleosomes [160]. In addition, BKRF4 has histone chaperone activity [161].

KSHV ORF45 is a tegument protein [12,18] with different functions to those of BKRF4 [162,163]. It is involved in viral replication [164]; assembly/envelopment/egress [165,166]; nucleocapsid transport [167]; and modification of the IRF7 [168,169], inflammasome [170], ERK/RSK [171,172], eIF4B [173], USP7/P53 [174,175], and ATF4 [176] signaling pathways. Modification of these signaling pathways by KSHV ORF45 is reminiscent of EBV BGLF2, but not their counterpart in EBV, BKRF4.

### 4.10 BLRF2 (homolog of KSHV ORF52)

The EBV BLRF2 gene encodes a gamma-herpesvirus-specific tegument protein expressed with late kinetics. The protein localizes to the nucleus and nuclear rim in transfected or infected cells [22]. Viral gene expression and viral DNA replication were not affected by the BLRF2 knockout, but production of infectious progeny into the culture medium was mildly but significantly dropped, possibly because of decreased infectivity [22]. A dimerization motif in the middle of BLRF2 mediates its self-association [177] and formation of this homodimer enhances protein stability [22]. The C-terminus of BLRF2 has a motif for phosphorylation by SRPK2, which is linked to progeny production [178]. Interestingly, BLRF2 also associates with many other tegument proteins and, thus, is likely to be a hub protein [22].

The KSHV tegument protein ORF52 is expressed with late kinetics. Its null mutant showed reduced virion production and infectivity [179]. Importantly, the null mutant failed to pack other tegument proteins, including ORF33 and ORF45 [179]. KSHV ORF52 antagonizes the host DNA sensor cGAS, thereby promoting immune evasion [[180], [181], [182]]. ORF52 homologs in other gamma-herpesviruses, including EBV BLRF2, inhibit the antiviral response [182]. ORF52 is structurally similar to the HSV tegument protein VP22 (UL49) (Fig. 2), and both are involved in microtubule reorganization and immune evasion [[183], [184], [185], [186]], suggesting that non-homologous genes in alpha- and gamma-herpesviruses encode products with similar functions.

### 4.11 BGLF3.5 (homolog of HSV UL14 and KSHV ORF35)

EBV BGLF3.5 is conserved in alpha- and gamma-, but not in beta-, herpesviruses [187]. BGLF3.5 associates with other tegument proteins, such as BSRF1 and BLRF2. BGLF3.5 is likely to be a tegument gene, although its product has not been identified in EBV virion, possibly because of its small size. Knockout of BGLF3.5 did not affect virus replication or progeny production in HEK293 cells [188].

HSV UL14 is a homologous tegument protein [189] that increases the progeny titer by about one order of magnitude [16,190]. It has multiple functions, such as chaperone-like activity [191], protection from apoptosis [192], and aiding nuclear transport of VP16 and nucleocapsids [190,193]. KSHV ORF35 was detected in purified virions [18]. Knockout of ORF35 in KSHV slightly decreased viral gene expression, viral DNA synthesis, and progeny titer compared to the wild-type [194]. In MHV-68, viral gene expression and viral DNA synthesis were unaffected, but production of infectious progeny was decreased by the knockout [195].

### 4.12 BALF1 (possibly related to KSHVORF16)

EBV encodes two homologs of the host anti-apoptotic protein BCL2: BHRF1 and BALF1 [196,197]. Knockout of either or their simultaneous disruption had little effect on virus multiplication and progeny production. However, knockout of either moderately decreased B cell transformation efficiency, and their simultaneous disruption almost abolished B cell transformation [198], indicating that both vBCL2 proteins are required for B cell transformation. BALF1 reportedly stimulates autophagy [199]. We reported that it interacts with BSRF1 and is incorporated into the tegument [14].

KSHV has one BCL2 homolog, ORF16, the absence of which decreases viral gene expression, DNA synthesis, and progeny production [200]. Intriguingly, KSHV ORF16 protein interacts with ORF55, a homolog of the EBV tegument protein BSRF1, and mediates the incorporation of tegument proteins into virions [15].

### 4.13 BALF2 (homolog of HSV UL29, HCMV UL57, and KSHV ORF6)

BALF2 encodes a single-stranded DNA-binding protein essential for lytic viral DNA synthesis [201,202], and has been detected in purified EBV virions [10]. Some BALF2 proteins localize to the cytoplasm during secondary envelopment [203]. BALF2 interacts with a CATC component, BVRF1 [203], which may mediate the transport of BALF2 protein to newly formed nucleocapsids. The HSV and KSHV homologs of BALF2, UL29 and ORF6, respectively, have also been detected in purified virions [18,189,204].

### 4.14 BMRF1 (homolog of HSV UL42, HCMV UL44, and KSHV ORF59)

Like BALF2, the BMRF1 protein of EBV is required for viral DNA replication [201,205]. BMRF1 increases the activity of the DNA polymerase catalytic subunit, BALF5 [206]. BMRF1 also activates transcription [[207], [208], [209]]. BMRF1 associates with BKRF4 and BLRF2 [22], and possibly with BGLF4, by which it is phosphorylated [128]. EBV BMRF1 and HSV UL42 proteins were detected in purified virions [10,189], but KSHV ORF59 was not [204].

### 4.15 BXLF1 (homolog of HSV UL23 and KSHV ORF21)

BXLF1 encodes a thymidine kinase (TK), which is a component of the thymidine salvage pathway of nucleotide biosynthesis [210]. Disruption of the gene had little effect on virus multiplication in cell culture [211]. The protein has been detected in EBV particles [10], but its importance is unknown.

The alpha- and gamma-herpesviruses, but not the beta-herpesviruses, have TK genes. HSV TK (UL23) and KSHV TK (ORF21) have been detected in the virion tegument fraction [18,189,204]. Disruption of HSV UL23 moderately decreased virus replication [212]. Knockout of KSHV ORF21 had no effect on viral gene expression and DNA synthesis, but markedly suppressed infectious progeny production, presumably by decreasing infectivity [213]. Upon infection, ORF21 protein in the tegument is released into the cytoplasm, where it stimulates MEK signaling to enhance infectivity.

### 4.16 BORF2 (homolog of UL39, HCMV UL45, and KSHV ORF61)

The BORF2 gene of EBV encodes the large subunit of ribonucleotide reductase (RR), which is involved in nucleotide biosynthesis. A BORF2-null mutant virus exhibited lower progeny production than the wild-type [214]. Although the physiological role of BORF2 in the tegument is unknown, expression of the BORF2 gene increases the proportion of cells at G_1_/S phase by increasing the P53 protein level [108]. In addition, BORF2 associates with, and thereby suppresses the activity of, APOBEC3B, to maintain genomic integrity [214,215], which increases the number of cells in G_1_/S phase [216].

The large subunit of the RR gene is preserved in HSV, CMV, and KSHV, but HCMV UL45 lacks enzymatic activity [217]. HSV UL39 is non-essential for multiplication in cell lines but required for efficient replication in non-dividing cells [218]. EBV BORF2, HSV UL39, and KSHV ORF61 inhibit the restriction factor, APOBEC3B [219]. HCMV UL45 [220] and HSV UL39, but not KSHV ORF61 protein, have been detected in virions [204], together with the RR small subunit [189]. Two-hybrid analysis indicated that HCMV UL45 protein serves as a tegument network hub, and has many interactions [221].

### 4.17 BRLF1 (homolog of KSHV ORF50)

BRLF1 is one of the two IE genes encoded by EBV implicated in viral reactivation. This transcriptional activator is required for lytic initiation, particularly in differentiated epithelial cells [222]. BRLF1 protein is incorporated into the tegument [19] and binds a capsid triplex protein, BORF1, thereby preventing its ubiquitin-dependent degradation in transfected cells [19].

Homologs of EBV BRLF1 are found only in gamma-herpesviruses. KSHV ORF50, also known as K-Rta, is a key lytic activator [223], which, unlike EBV BRLF1, has not been detected in purified virions [204].

### 4.18 BRRF2 (homolog of KSHV ORF48)

The BRRF2 gene product is a late phosphoprotein. BRRF2 localizes to the cytoplasm and its knockout decreased progeny production [224]. Its homologs are present only in gamma-herpesviruses. KSHV ORF48, a homolog of BRRF2, was detected in virions [18], but its function is unclear.

## 5 Roles of EBV tegument proteins in oncogenesis

There are at least 10 hallmarks of cancer—evasion of apoptosis, self-sufficiency in growth signals, insensitivity to anti-growth signals, tissue invasion and metastasis, limitless replicative potential, sustained angiogenesis, dysregulation of cellular energetics, avoidance of immune destruction, tumor-promoting inflammation, and genome instability and mutation [225]. Latent genes of EBV, such as LMP1, LMP2A, EBNA1, EBNA2, EBNA3A, EBNA3C, and some viral microRNAs, have oncogenic activity, but accumulating pieces of evidence indicate that lytic genes are also involved in oncogenesis [[226], [227], [228], [229]]. Below we summarize the EBV genes linked to oncogenesis.

EBV has two BCL2 homologs, BHRF1 and BALF1, that contribute to evasion of apoptosis [198], and BALF1 may be present in the tegument [14]. Self-sufficiency in growth signals is conferred mainly by latent genes, LMP1 and LMP2A, which activate CD40 and BCR signaling, respectively, in a ligand-independent manner [230]. BZLF1 or other viral factors, like LMP1, induce the production of chemokines and cytokines (such as TNF-α, IL-6, IL-8, VEGF, and IL-10) that activate growth signals [[231], [232], [233]]. BGLF2 and BRLF1 induce MAPK signaling [109,110,234]. Insensitivity to anti-growth signals includes overcoming cell-cycle checkpoints by disabling proteins such as P53, and CDK inhibitors. The latent genes, LMP1, EBNA1, and EBNA3C have been reported to deregulate P53 [235,236]. Transcription of CDK inhibitors, such as P16, P21 and P27, is repressed by the latent genes EBNA3A and EBNA3C [[237], [238], [239], [240]]. Having CDK-like kinase activity, BGLF4 phosphorylates and inactivates P27 [131] and RB [241]. Regarding tissue invasion and metastasis, the EBV genes LMP1, LMP2A, EBNA1, EBNA3C, and BZLF1 induce the epithelial-to-mesenchymal transition and the emergence of cancer stem cells, and encourage migration and invasion [[242], [243], [244], [245], [246]]. Maintenance of telomere length by telomere reverse transcriptase (TERT) is linked to limitless replicative potential. EBV LMP1 induces the TERT gene and maintains telomere synthesis [247,248]. LMP1, LMP2A, BZLF1, and BRLF1 induce pro-angiogenic factors, including VEGF and FGF, thereby mediating sustained angiogenesis [[249], [250], [251], [252], [253]]. The EBV latent gene, LMP1, causes deregulation of cellular energetics, increasing glycolysis and lactate production by inducing HK2, PKM2, LDHA1, and GLUT1 [251,[254], [255], [256], [257]]. EBV encodes many latent and lytic genes linked to avoidance of immune destruction [258,259]. For instance, EBNA2, BZLF1, BGLF5, BILF1, and BNLF2A downregulate antigen presentation by HLA [[260], [261], [262], [263], [264], [265]]. EBNA2 and LMP1 repress the expression of PD-L1 [266]. Innate immunity is repressed by several latent and lytic proteins, including EBNA1, EBNA2, LMP1, LMP2A, BGLF5, BPLF1, BOLF1, BGLF4, BRLF1, BGLF2, and BCRF1 (vIL-10) [86,112,113,137,[267], [268], [269], [270], [271], [272], [273], [274], [275], [276]]. Intrinsic immune mechanisms, such as epigenetic silencing and PML-NB-mediated suppression, are reversed by EBNA1, EBNA2, EBNA3, BZLF1, BRLF1, and BNRF1 [156,[277], [278], [279], [280]]. With respect to tumor-promoting inflammation, the production of cytokines and chemokines is induced by EBV genes, as mentioned above. Finally, the EBV genes BZLF1, BORF2, and BGLF4 dysregulate P53 and CDK inhibitors, leading to genome instability and mutation [139,281,282]. LMP1, EBNA1, EBNA2, EBNA3C, BRLF1, BNRF1, BKRF4, BPLF1, and BGLF5 disrupt genomic integrity [157,159,281,[283], [284], [285], [286], [287], [288]].

## 6 Conclusion

We summarized the interactions and roles of EBV tegument proteins. The tegument proteins of the Herpesviridae have several conserved properties and functions whereas others are unique to EBV. The phenotypes of EBV knockout mutants are typically analyzed in HEK293 cells, but some processes of the EBV lifecycle are difficult to evaluate in the cell line, *e.g.*, secondary envelopment. This may be because EBV replication is less efficient than HSV, and nucleocapsids cannot be easily detected in HEK293 cells. Alternatively, HEK293 cells might be conducive to secondary envelopment even in the absence of one or more tegument genes. BSRF1 knockout virus had a phenotype similar to wild-type virus in HEK293 cells, but knockdown of the BSRF1 gene reduced progeny virus production in B95-8 cells [14]. Therefore, EBV gene functions should be analyzed in the virus's natural host cell type, such as B cells, for further evaluation.

EBV tegument genes are good candidates for viral attenuation, for development of live vaccines, because their disruption does not cause complete inactivation and so does not prevent the production of other viral antigens, *e.g.*, glycoproteins. Such development requires detailed analyses of knockout viruses, including in animal models.

## Author statement

**Takayuki Murata**: Conceptualization; Data curation; Funding acquisition; Project administration; Supervision; Validation; Visualization; Writing - original draft; Writing - review & editing.

## Funding sources

This work was supported by 10.13039/100009619Japan Agency for Medical Research and Development (JP21wm0325042) and the 10.13039/100007449Takeda Science Foundation.

## Declaration of competing interest

The authors declare that they have no known competing financial interests or personal relationships that could have appeared to influence the work reported in this paper.

## Data Availability

No data was used for the research described in the article.
